# CD14 Is a Co-Receptor for TLR4 in the S100A9-Induced Pro-Inflammatory Response in Monocytes

**DOI:** 10.1371/journal.pone.0156377

**Published:** 2016-05-26

**Authors:** Zhifei He, Matteo Riva, Per Björk, Karl Swärd, Matthias Mörgelin, Tomas Leanderson, Fredrik Ivars

**Affiliations:** 1 Immunology group, Section for Immunology, Department of Experimental Medical Science, Lund University, Lund, Sweden; 2 Active Biotech AB, Lund, Sweden; 3 Section for Cell and Tissue Biology, Department of Experimental Medical Science, Lund University, Lund, Sweden; 4 Section for Infection Biology, Department of Clinical Sciences, Lund University, Lund, Sweden; INSERM, FRANCE

## Abstract

The cytosolic Ca^2+^-binding S100A9 and S100A8 proteins form heterodimers that are primarily expressed in human neutrophils and monocytes. We have recently shown that S100A9 binds to TLR4 in vitro and induces TLR4-dependent NF-κB activation and a pro-inflammatory cytokine response in monocytes. In the present report we have further investigated the S100A9-mediated stimulation of TLR4 in monocytes. Using transmission immunoelectron microscopy, we detected focal binding of S100A9 to monocyte membrane subdomains containing the caveolin-1 protein and TLR4. Furthermore, the S100A9 protein was detected in early endosomes of the stimulated cells, indicating that the protein could be internalized by endocytosis. Although stimulation of monocytes with S100A9 was strictly TLR4-dependent, binding of S100A9 to the plasma membrane and endocytosis of S100A9 was still detectable and coincided with CD14 expression in TLR4-deficient cells. We therefore investigated whether CD14 would be involved in the TLR4-dependent stimulation and could show that the S100A9-induced cytokine response was inhibited both in CD14-deficient cells and in cells exposed to CD14 blocking antibodies. Further, S100A9 was not internalized into CD14-deficient cells suggesting a direct role of CD14 in endocytosis of S100A9. Finally, we could detect satiable binding of S100A9 to CD14 in surface plasmon resonance experiments. Taken together, these results indicate that CD14 is a co-receptor of TLR4 in the S100A9-induced cytokine response.

## Introduction

It is well established that both intracellular proteins, as well as fragments of extracellular proteins released upon tissue injury, can become ligands mediating sterile inflammation (reviewed in [1–4]). Such proteins are denoted damage associated molecular patterns (DAMPs). Binding of DAMPs to receptors such as TLR4 or RAGE, has been shown to induce the production of pro-inflammatory cytokines both in immune cells such as dendritic cells and macrophages as well as in other tissue resident cells.

S100 proteins are low molecular weight Ca^2+^ binding proteins, which are expressed in a tissue-specific manner in various cells of the human body (reviewed in [5–7]). Most of these proteins reside in the cytosol of the cells, while some are secreted. The S100A9 protein is normally expressed as a heterodimer together with the S100A8 protein in myeloid cells. In human cells, these proteins are co-expressed both in neutrophils and monocytes/macrophages [8–11], while in the mouse they are mainly expressed in neutrophils. The S100A8/A9 heterodimer is highly abundant in human neutrophils and constitutes a large part of the total protein content of these cells [9, 10].

The S100A8/A9 heterodimer can be secreted by activated monocytes [12], but the molecular mechanism of secretion is still largely unknown. Further, these proteins are released in high amounts by neutrophils during various inflammatory conditions and can be used as markers of inflammation (reviewed in [13–15]). In the extracellular milieu, both the S100A8 [16–19] and S100A9 proteins [17, 18, 20–22] have been reported to possess pro-inflammatory function and are therefore considered to be damage associated molecular patterns (DAMPs). Thus, both huS100A8 [19, 23] and huS100A9 [21, 24] interact with TLR4 and stimulate production of pro-inflammatory cytokines in monocytes. Interestingly, we also found that moS100A9 could induce activation of inducible nitric oxide synthase (iNOS) expression in bone marrow-derived dendritic cells (BM-DCs). That finding suggested to us that S100A9 could also stimulate the endosomal pathway of TLR4 stimulation involving activation of β-interferon (IFNβ) expression [25]. The activation of iNOS expression by S100A9 would, in analogy with the response to LPS [25], be expected to involve endocytosis of the S100A9/TLR4 complex in the responding monocytes.

The mechanism of activation of TLR4 by bacterial lipopolysaccharide (LPS) and the downstream intracellular signaling pathways has been extensively investigated [26, 27]. Seminal studies by Beutler and coworkers demonstrated that TLR4 was the LPS-receptor [28]. Subsequently reports from several laboratories showed that binding of LPS also involved the LPS binding protein MD2 and CD14 [29–31]. CD14 is a glycosylphosphatidylinositol-anchored membrane protein [32], which has several functional roles in the LPS-induced stimulation of TLR4. First, and most importantly, CD14 is essential for the internalization of the LPS/TLR4 complex by endocytosis [33] and hence for the IFNβ response induced by the cytosolic TRIF/TRAM-dependent pathway of TLR4 activation [34]. Second, CD14 binds LPS and enhances the LPS-responsiveness by TLR4/MD2 [35]. Interestingly, CD14 is also involved in the internalization of TLR3 and enhances signaling from that receptor [36].

The role of CD14 in DAMP-induced TLR4 stimulation has also been investigated previously (reviewed in [4]), but the functional role of the CD14 protein is not fully understood. In this report we have further investigated the mechanism of the S100A9-induced stimulation of the TLR4-dependent cytokine response in monocytes. We show that TLR4 is neither essential for the binding for S100A9 to the plasma membrane nor for the internalization of S100A9 into these cells. Further, we present findings indicating that S100A9 can bind CD14 and that CD14 is an essential co-receptor for S100A9-mediated TLR4-stimulation.

## Materials and Methods

### Mice

C57BL/6 mice were bought from Taconic Europe (Ry, Denmark). TLR4-KO and CD14-KO mice were originally bought from Jackson Laboratories (Bar Harbor, MN USA). Caveolin-1 deficient (cav-1-KO) mice [37] were bred onto C57BL/6 genetic background [38]. TLR4-KO and cav-1-KO mice were bred in the animal facilities of the Biomedical Center at Lund University. All experiments in this study involving the use of cells from animals were approved of by the local ethics committee of animal experiments of Malmö and Lund (permit M12/13).

### Cell culture

The human monocytic leukemia cell line THP-1 (from ATCC, Mannassas, VA, USA) was cultured in RPMI-1640 medium (Invitrogen, Stockholm, Sweden) supplemented with 10% fetal bovine serum (Invitrogen), 2 mM glutamine, 1 mM sodium pyruvate, 10 mM HEPES, 100U/ml penicillin and 100μg/ml streptomycin (all supplements from Invitrogen). Bone marrow derived dendritic cells (BM-DCs) were prepared by culturing cells from femurs and tibias of mice in the same medium as above. The medium was supplemented with 10% tissue culture supernatant of J558L cells transfected with a GM-CSF cDNA construct and cells were harvested on day 7 of culture. Peritoneal wash cells from C57BL/6 mice were used in some experiments. For in vitro stimulation experiments cells were cultured in triplicate using the above medium and stimulated with huS100A9, Ultra-pure E.coli K12 LPS (InvivoGen, San Diego CA) or the synthetic lipopeptide Pam_3_Cys (EMC Microcollections GmbH, Tuebingen, Germany) as indicated in Figure legends.

### Cytokine assays

Supernatants were harvested from in vitro cultures and stored at -80°C until use. TNFα concentration was determined using the Cytokine Bead Array (CBA; BD Biosciences, San Jose California) and the human and mouse TNF Flex sets (BD Biosciences), according to the manufacturer’s protocols. Analysis was performed using an LSR II flow cytometer (BD Biosciences).

### Antibodies

For CD14 blocking experiments, mouse anti-human CD14 antibody (clone M5E2; Novus Biologicals, Littleton CO, USA) and rat anti-mouse (clone 4C1/CD14; BD Biosciences) were used. The following antibodies were used for staining specimens for electron microscopy: rabbit anti-caveolin 1 antibodies (BD Biosciences), goat anti-mouse TLR4 (M16) (Santa Cruz Biotechnology, CA USA), rabbit anti-Rab5 (ab18211, Abcam, Cambridge, UK) and rat anti-mouse CD14 (clone Sa14-2; Biolegend, Nordic Biosite, Täby, Sweden).

### Recombinant S100A9 proteins

Preparation of the human S100A9 (huS100A9) protein was described in detail in our previous paper [21] and purification of mouse S100A9 (moS100A9) was performed using the same protocol. Endotoxins were removed using Detoxi-Gel columns (Thermo Scientific). The endotoxin content was tested using LAL Chromogenic Endpoint assay (Hycult Biotechnology, Uden, The Nederlands). The huS100A9 and moS100A9 protein batches used in here contained 0.12 EU/ml and 0.036 EU/ml endotoxin, respectively. In experiments analyzing the biological activity of the proteins, polymyxin B was included in control cultures.

### Transmission electron microscopy

THP-1 cells or mouse BM-DCs were incubated with recombinant human S100A9 protein conjugated with colloidal gold (10nm particles) for 15 min at 37°C. The cells were harvested and washed once with PBS. Thereafter the cell pellet was re-suspended in 150mM sodium cacodylate/2.5% glutaraldehyde, pH 7.4 (EM-fix solution) and incubated at room temperature over night. Cells were then prepared for immunostaining and transmission electron microscopy as recently described [39]. Briefly, ultrathin sections of the cells were subjected to antigen retrieval with metaperiodate and then incubated over night at 4°C with primary antibodies in PBS (≈10 μg/ml). Binding of the primary antibodies was detected with secondary antibodies of appropriate specificity, conjugated with 25nm colloidal gold (Electron Microscopy Sciences, Fort Washington, PA, USA; titer 1:1 to 1:20). Specimens were examined in a Philips/FEI CM100 transmission electron microscope operated at 60 kV accelerating voltage. Images were recorded with a side-mounted Olympus Veleta camera with a resolution of 2048x2048 pixels (2Kx2K).

### Surface plasmon resonance (SPR) analysis

Binding of CD14 to recombinant human S100A8 and S100A9 (produced at Active Biotech AB, Lund, Sweden) was analyzed using the surface plasmon resonance (SPR) technology on a Biacore 3000^TM^ system (GE Healthcare, Uppsala, Sweden). Briefly, the S100 proteins were immobilized in separate flow cells on a CM5 chip through standard amine coupling. Then recombinant human CD14, derived from CHO cells (R&D Systems cat no 383-CD-050/CF), was injected (for 2 min at a flow rate of 30 μl/min) in 10 mM HEPES, 0.15 M NaCl, pH 7.4, containing 0.005% v/v surfactant P20 (HBS-P buffer) and 1 mM Ca^2+^ and 20 μM Zn^2+^. Regeneration was performed by a 30 μl pulse of 3 mM EDTA in HBS-P buffer. Resulting sensorgrams were fit to a 1:1 model using the BIAevaluation software 4.1.

## Results

### S100A9 binds to monocyte membranes and can be internalized by these cells

**Fig 1:**
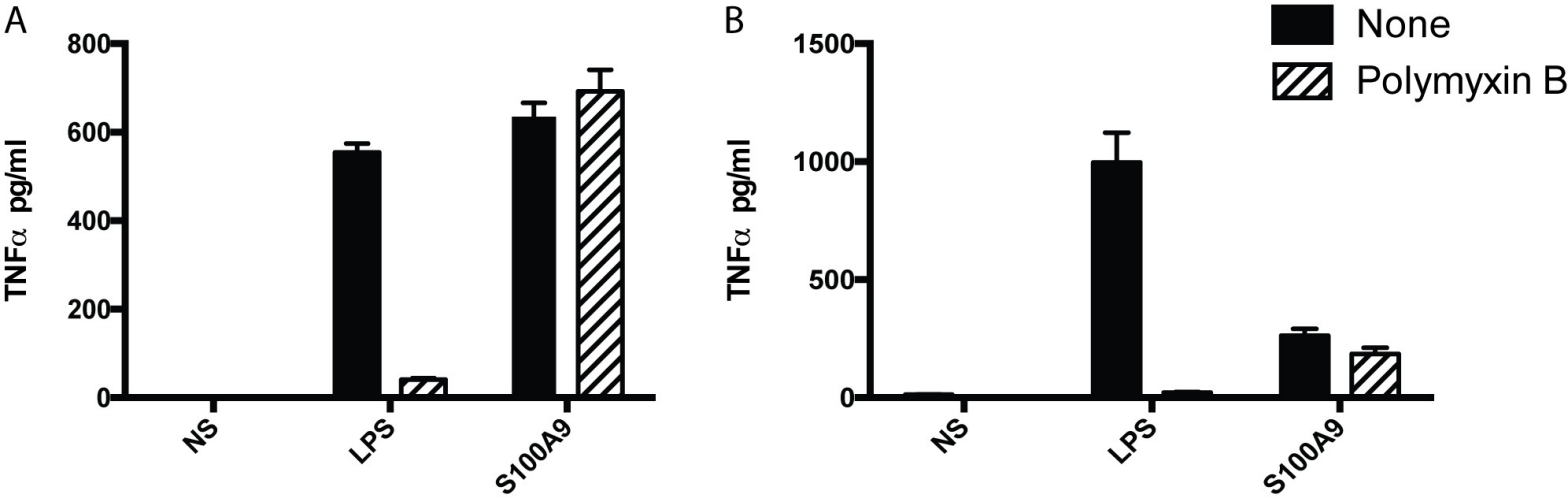
S100A9 induces a TNFα-response in monocytoid cells. (A) THP-1 cells (1x10^6^/ml) or (B) peritoneal wash cells (1x10^6^/ml) were stimulated either with huS100A9 (40μg/ml) or LPS (A, 100ng/ml; B, 10ng/ml), with or without pre-incubation with polymyxin B (50μg/ml) for 30min. Supernatants were harvested after (A) 4hrs or (B) 24 hrs of culture and TNFα concentration determined using CBA assay. Results are representative of 3 (A) and 2 (B) independent experiments.

We have previously shown, and confirm in here (**Fig 1A**) that the huS100A9 protein can induce a cytokine response in the human THP-1 monocyte cell line. Further, huS100A9 can stimulate a cytokine response also in mouse macrophages (**Fig 1B**). As expected, in both cases addition of polymyxin B strongly reduced the LPS-induced response. Importantly, this compound had little impact on the S100A9-induced response, indicating that putative LPS contamination of the recombinant protein preparation contributed minimally to the response.

**Fig 2:**
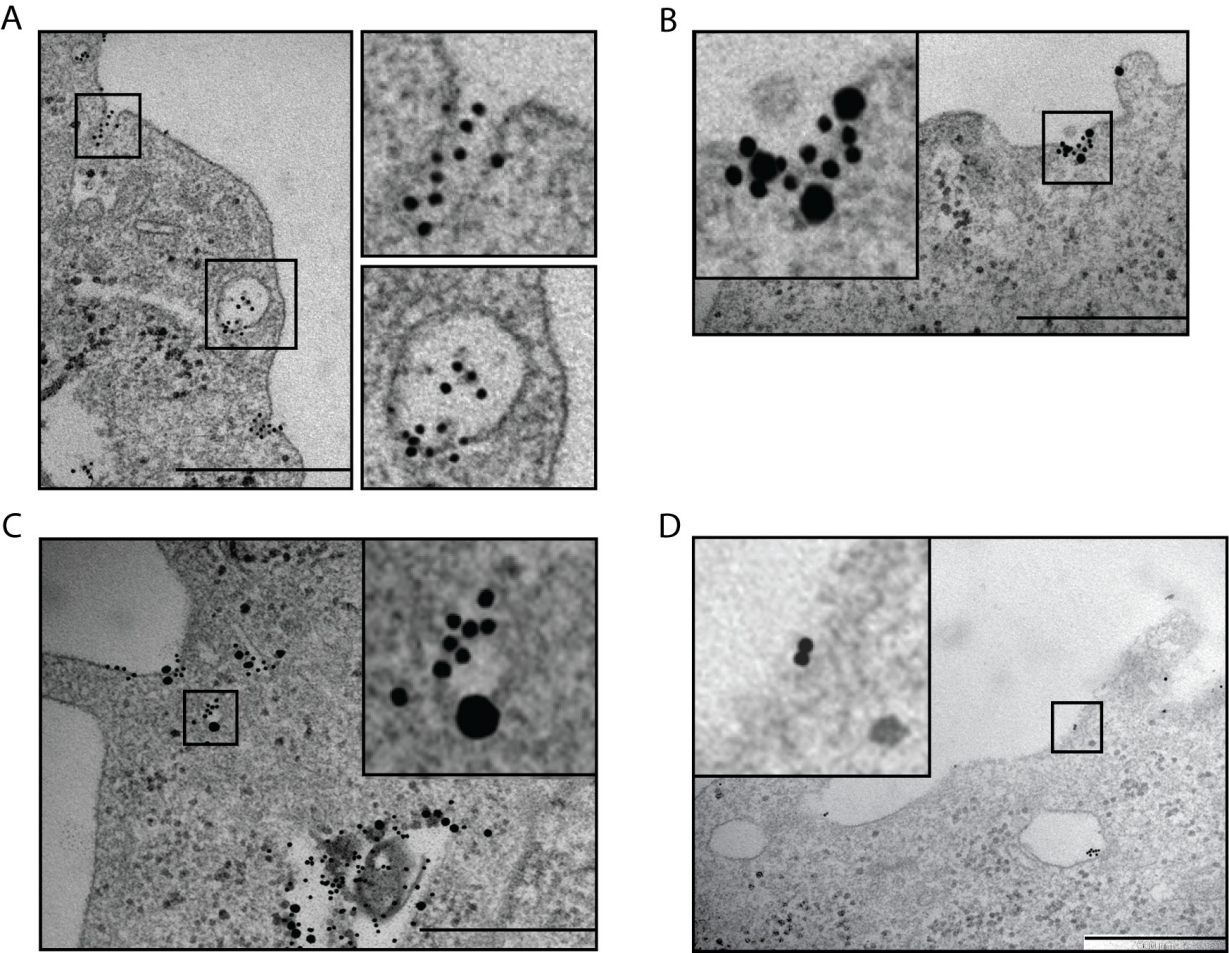
Binding of S100A9 and TLR4 expression coincide on monocyte cell surface. (A,B) THP-1 cells were incubated with 1μM colloidal gold-labeled S100A9 (10nm grains) protein for 15 min at 37°C and specimens prepared for TEM analysis. (B) Specimens from the same preparation were immuno-stained with TLR4 antibody, followed by secondary gold-labeled (25nm grains) anti-goat Ig antibody. The images show representative sites of surface and vesicular binding of the S100A9-protein and TLR4-expression. (C) Specimens from the preparation of THP-1 cells used in (A) were immuno-stained with Rab5 antibody conjugated with colloidal gold (25nm); bar 500nm. (D) THP-1 cells exposed to MβCD do not display focal S100A9 binding. THP-1 cells were cultured in presence of 15mM MβCD for 30 min, thereafter washed and incubated with colloidal gold-labeled S100A9 as in (A). Bar: 500nm.

Our previous data suggested that the S100A9-protein, similarly to LPS, might also be internalized into the responding cell upon TLR4 stimulation [21]. To address this possibility, we labeled huS100A9 with colloidal gold and incubated THP-1 cells with the gold-conjugated huS100A9. Transmission electron microscopy analysis revealed that huS100A9 displays focal binding in pit-like structures on the THP-1 cell surface (**Fig 2A**). Further, the protein was also detected in vesicles in the cytosol of these cells, suggesting that the protein had been internalized by endocytosis. Staining of specimens from these cells with TLR4 antibodies revealed similar focal co-localization of huS100A9 with TLR4 in pit-like structures (**Fig 2B**). Cytosolic vesicles in which huS100A9 co-localized with TLR4 (**S1A Fig**) as well as with the early endosomal marker Rab5 (**Fig 2C**) could also be detected in these cells. These data further support that stimulation with S100A9, similarly to stimulation with LPS [40, 41] may involve internalization of TLR4.

### S100A9 binds to caveolin-1 containing membrane subdomains

We next wanted to investigate the nature of the membrane subdomains with focal binding of S100A9 and accumulation of TLR4. Previously published findings indicate that upon stimulation of monocytes with LPS, both TLR4 and CD14 accumulate in membrane subdomains containing lipid rafts [42, 43]. Treatment of cells with methyl-β-cyclodextrin (MβCD) sequesters cholesterol and is known to interfere with lipid raft function [44, 45]. Interestingly, in MβCD-treated THP-1 cells there was only occasional binding of the protein to the plasma membrane (**Fig 2D**) and there was no focal binding similar to what was seen in untreated THP-1 cells (compare to **Fig 2A**).

**Fig 3:**
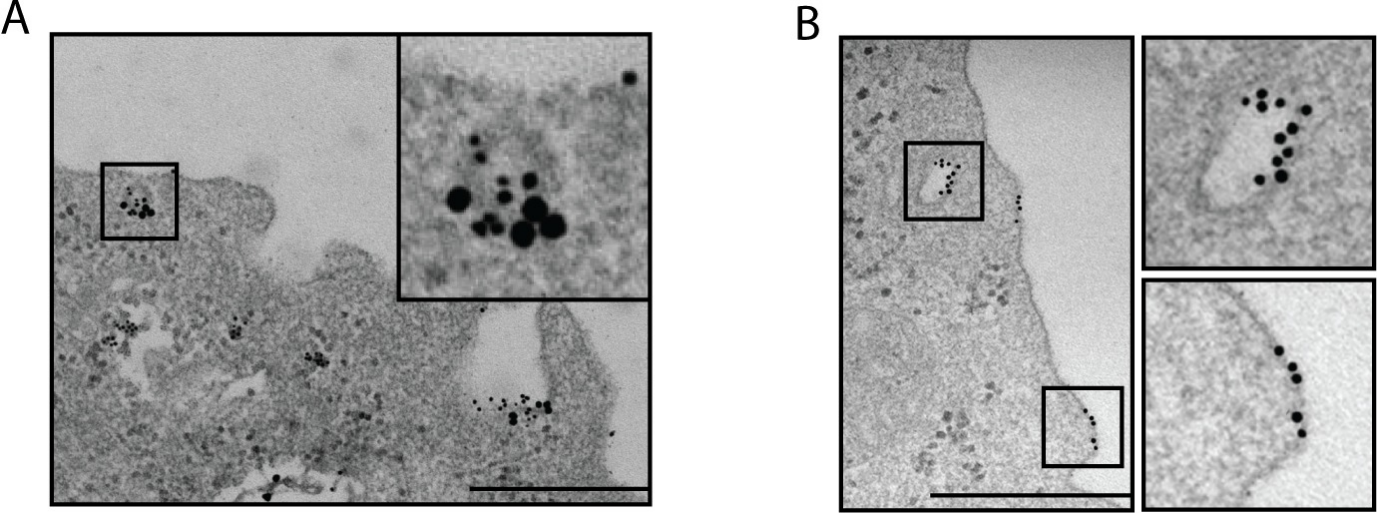
S100A9 binds focally both at membrane sites expressing and lacking cav-1. BM-DCs from (A) C57BL/6 or B) cav-1-KO mice were incubated with gold-labeled S100A9 (10nm grains) as in Fig 2. (A) The specimen was thereafter immuno-stained with rabbit anti-cav-1 antibody, followed by gold-labeled (25nm grains) secondary antibody. The images show representative sites of surface and vesicular binding of the S100A9-protein and TLR4-expression. Bars: 500nm.

Both monocytes and DCs express the protein caveolin-1 (cav-1), which is known to associate with caveolae-like membrane subdomains in such cells (reviewed in [46]). We therefore stained specimens of S100A9 exposed mouse BM-DCs with caveolin-1 specific antibodies. As can be seen (**Fig 3A**), S100A9 bound to similar membrane subdomains in the BM-DCs and the sites of focal S100A9 binding co-localized with cav-1 staining. Interestingly, we could also detect occasional binding of S100A9 to the membranes of caveolin-1 deficient (cav-1^-/-^) BM-DCs and the protein could also be internalized by these cells (**Fig 3B**). These data taken together suggest that S100A9 binds to membrane subdomains containing lipid rafts and cav-1, but that presence of cav-1 is not essential for the binding and internalization.

### TLR4-independent internalization of S100A9 in BM-DCs

**Fig 4:**
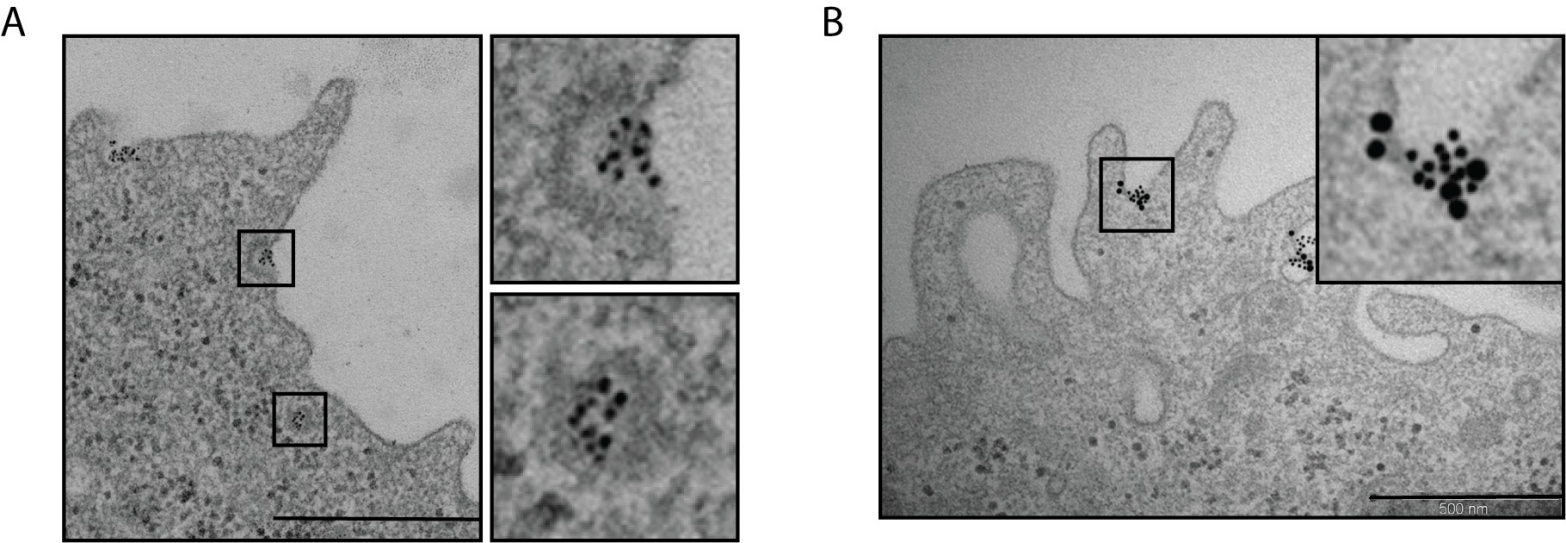
Focal S100A9 membrane binding and internalization is TLR4-independent and coincides with Cav-1 expression. (A,B) BM-DCs from TLR4-KO mice were incubated with gold-labeled S100A9 (10nm grains) as in Fig 2. (B) The specimen was thereafter immuno-stained with rabbit anti-cav-1 antibody, followed by gold-labeled (25nm grains) secondary antibody. The images show representative sites of surface and vesicular binding of the S100A9-protein and cav-1-expression. Bars: 500nm.

The above data indicated that S100A9 could be internalized into BM-DCs by endocytosis. While, as shown in our previous report [21], TLR4 expression was essential for the S100A9-induced cytokine response in BM-DCs, we also wanted to know whether TLR4 would be required for the internalization of S100A9. TEM analysis of TLR4-KO BM-DCs incubated with gold-labeled S100A9 revealed focal binding of S100A9 to the plasma membrane (**Fig 4A**). The finding that S100A9 was also detected in cytosolic vesicles of these cells, suggested that internalization of this protein is TLR4-independent. Also in the TLR4-KO cells, the focal binding to the plasma membrane (**Fig 4B**) as well as S100A9-binding in cytosolic vesicles **(S1D Fig)**, co-localized with cav-1 expression. These data indicate that S100A9 can be internalized at cav-1 containing membrane subdomains through a TLR4-independent mechanism.

### CD14 is a co-receptor of TLR4 in the S100A9-induced cytokine response

The above data indicated that there is at least one cell membrane associated receptor molecule that can bind and internalize S100A9 even in the absence of TLR4. Previous reports have described the involvement of CD14 in the internalization of LPS and an essential role of this protein in the LPS-induced IFNβ-response [33, 47]. We therefore next wanted to address whether CD14 might also be involved as a co-receptor in the S100A9-induced response. The observation that the focal binding of S100A9 to TLR4-KO BM-DCs co-localized with focal CD14 staining provided support for this possibility (**S2A Fig**).

**Fig 5:**
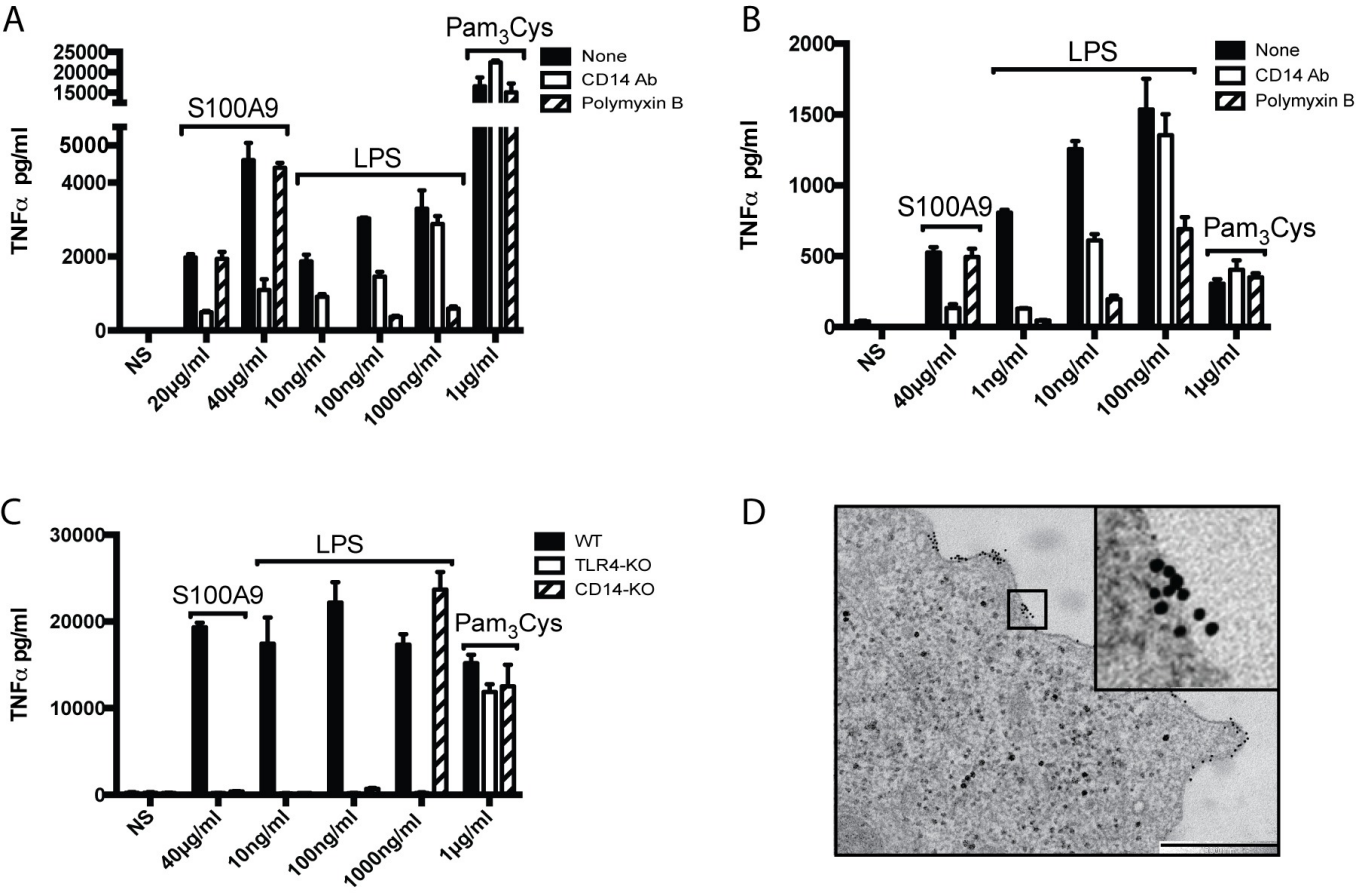
CD14 is essential for the S100A9-induced cytokine response. (A,B) Blocking of CD14 inhibits S100A9-induced cytokine response. (A) THP-1 cells (1x10^6^/ml) or (B) peritoneal wash cells (1x10^6^/ml) were pre-incubated with (A) mouse-anti-human CD14 blocking antibody (5μg/ml), (B) rat-anti-mouse CD14 blocking antibody (10μg/ml), (A,B) polymyxin B (50μg/ml) or medium (NS) for 30min, as indicated. The cells were subsequently cultured in medium (NS) or stimulated with indicated concentration of S100A9 protein, LPS or TLR2-stimulator Pam_3_Cys (1μg/ml); cells were simulated with 40μg/ml S100A9 in (B). Supernatants were harvested after (A) 4hrs or (B) 24 hrs of culture and TNFα concentration determined using CBA assay. Results from one representative experiment out of 3 (A) and 2 (B) independent experiments are shown. (C) BM-DCs from indicated mouse strains were stimulated with 40μg/ml moS100A9, or with LPS or Pam_3_Cys for 24 hrs and culture supernatants analyzed as in (A,B). Results are representative of two experiments. (D) CD14-KO DCs were incubated with gold-labeled S100A9 (10nm grains) and analyzed as in Fig 2. Bar: 500nm.

To determine whether CD14 would be involved in the S100A9-induced cytokine response we used an antibody known to block the interaction between LPS and CD14. This antibody readily reduced the S100A9-induced TNFα response in THP-1 cells (**Fig 5A**). We also confirmed that the antibody could block the TNFα response induced by a low concentration of LPS. This blockade, consistently with previously published data [33, 48], could be overcome by increasing concentrations of LPS. As expected, the antibody did not interfere with the TLR2-mediated Pam_3_Cys-induced response. We obtained similar results when mouse peritoneal macrophages were stimulated with S100A9 in the presence of an antibody that blocks binding of LPS to mouse CD14 **(Fig 5B)**. To confirm these data we performed similar stimulation experiments using mouse BM-DCs. As can be seen, while the S100A9 protein induced a robust TNFα-response in wt BM-DCs, the response was strongly reduced in CD14-KO BM-DCs (**Fig 5C).** The response was also strongly reduced in TLR4-KO BM-DCs, thereby confirming our previous data [21]. The addition of polymyxin B to the cultures **(Fig 5A and 5B and S2B Fig)** had only limited effect on the TNFα-response. Taken together, these data indicate that the S100A9-induced TNFα-response is both TLR4- and CD14-dependent.

While the TNFα-response was CD14-dependent, we could still detect S100A9 binding to the plasma membrane of both CD14-KO BM-DCs (**Fig 5D**) and TLR4-KO BM-DCs that had been pre-incubated with the CD14 blocking antibody (**S2C Fig**). These data suggest that there are also other S100A9-binding membrane molecule(s) except CD14 and TLR4 on these cells. However, there was no internalization of S100A9 neither in the CD14-KO BM-DCs nor in cells BM-DCs exposed to the blocking anti-CD14 antibody. Taken together, these data indicate that CD14 is an essential co-receptor in the S100A9-induced cytokine response and suggest that CD14 may also be essential for S100A9-internalization.

**Fig 6:**
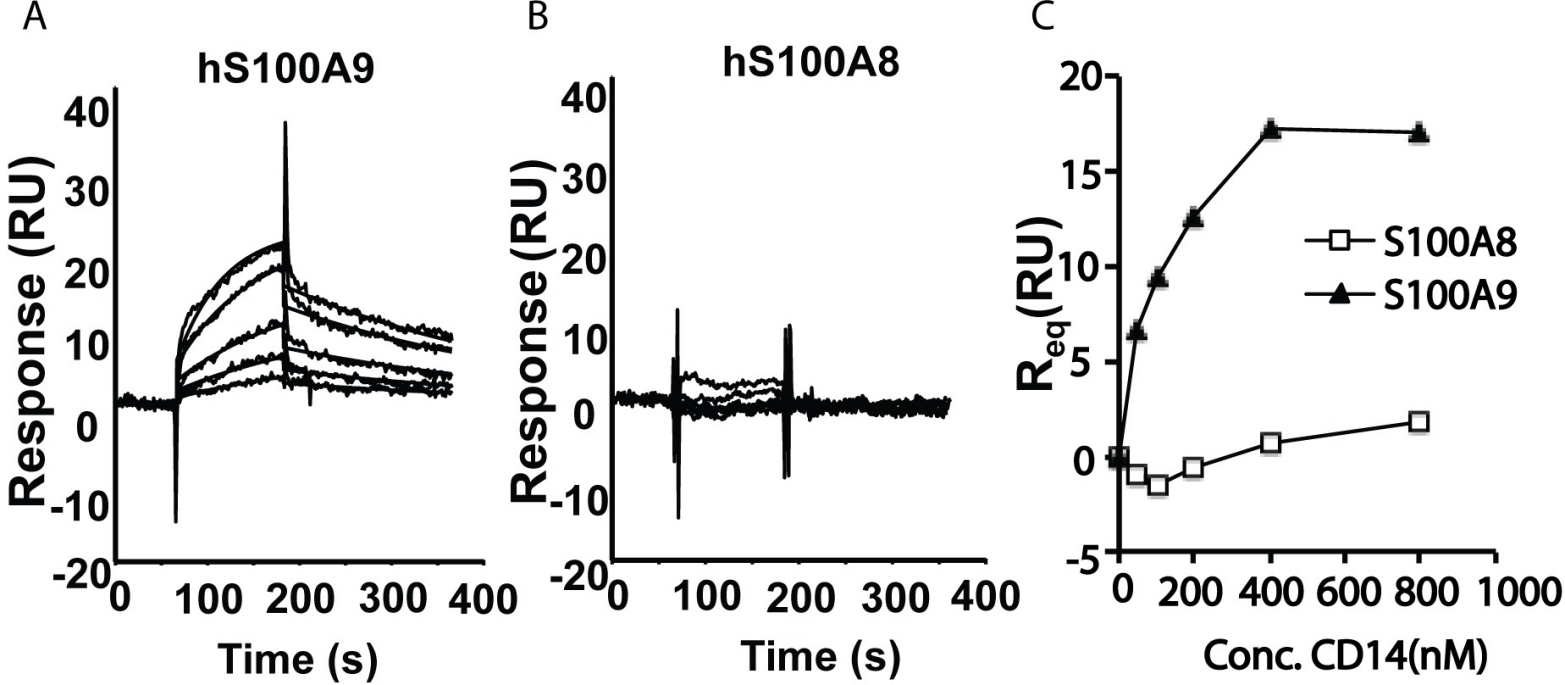
Interaction of CD14 with immobilized S100A8 and S100A9 using SPR analysis. Sensorgrams from bottom to top were obtained after injection of 50 to 800 nM CD14 over S100A9 (A) or S100A8 (B). Responses at steady-state (R_eq_) were calculated either by fit of sensorgrams to a 1:1 model (S100A9) or at late association phase (t 175 s) due to too fast kinetics (S100A8) and plotted vs CD14 concentration in C. A K_D_ of 0.1 to 0.2 μM was calculated for CD14 binding to S100A9 whereas non-satiable binding was obtained to S100A8. The data shows representative results from one out of two experiments performed.

SPR analysis was used to investigate whether human CD14 could directly interact with human S100A9. For this purpose, human S100A8 and S100A9 were immobilized to the same level on a chip and CD14 was passed over these surfaces. **Fig 6** shows sensorgrams obtained after injection of 50 to 800 nM CD14 over S100A9 (**Fig 6A**) or S1008 (**Fig 6B**). As is shown in **Fig 6C**, CD14 demonstrated satiable binding only to S100A9 with an affinity of 0.1 to 0.2 μM calculated after kinetic analysis of sensorgrams using a 1:1 model, whereas binding of CD14 to S100A8 was low and non-satiable in the concentration range used. Taken together, these data support the hypothesis that CD14 is an essential co-receptor for S100A9-induced TLR4-stimulation.

## Discussion

In this report we have investigated the mechanism of S100A9-induced TLR4 stimulation. We show by TEM analysis that the S100A9 protein, which is capable of inducing a TLR4-dependent TNFα-response in monocytes, displays focal binding to the plasma membrane of such cells. Previous studies from other laboratories had shown that the cav-1 protein is expressed in THP-1 cells [49, 50]. Analysis of plasma membrane from THP-1 cells induced to differentiate to macrophages, revealed that cav-1 associated with detergent resistant membrane domains i.e. lipid rafts [51]. Lipid rafts are membrane subdomains involved in signaling (reviewed in [52, 53]) that can be found in caveolae and are known to be involved in TLR4-stimulation (reviewed in [54]). Such cav-1-associated membrane subdomains in monocytoid cells thus most probably represent caveolae (reviewed in [46, 55]).

We speculated that the focal binding of the S100A9 protein might represent binding to membrane subdomains. It is known that TLR4 is recruited into lipid rafts upon stimulation of monocytoid cells with LPS [43] and that recruitment is reactive oxygen species dependent [56]. We could show that the sites of focal S100A9-binding on THP-1 cells coincided with focal expression of both TLR4 and the cav-1 protein. These results are consistent with a model according to which the stimulation of cells with the S100A9 protein via TLR4 would induce the recruitment of both TLR4 and S100A9 into lipid raft/caveolar membrane subdomains. The lack of focal S100A9-binding in MβCD-treated THP-1 cells was consistent with this model. In contrast, other investigators have reported that THP-1 cells lack caveolae-like membrane structures and have proposed that the TLR4-ligand LPS would be internalized by macropinocytosis [57, 58]. In these reports however, cav-1 expression was not investigated and these structures may therefore not have been detectable. We show in here that the S100A9 protein also binds to the plasma membrane of cav-1-KO BM-DCs and the protein could be detected in cytosolic vesicles of such cells. These data suggest that caveolae are neither essential for the binding nor for the internalization of the S100A9 protein. We speculate that the binding seen in these cells might reflect binding to S100A9 receptors located in lipid rafts. That hypothesis would be consistent with the only occasional, non-focal membrane binding detected in MβCD-treated cells.

Further, TLR4 is important for the LPS-induced inflammatory response but not for the internalization of LPS [59, 60]. In consistency with those findings, we previously showed [21] and confirmed in here that TLR4 is essential for the S100A9-induced TNFα-response in BM-DCs. In addition, we could also detect focal S100A9-binding to the plasma membrane of TLR4-KO BM-DCs and internalization of the protein in these cells. Previous studies in this field have shown that CD14 acts as a co-receptor during LPS-induced TLR4 stimulation [34]. Upon stimulation, CD14 plays important roles both for the recruitment of the TLR4/MD2/LPS complex into lipid raft membrane subdomains [43, 56] and for the internalization of that complex through endocytosis [33, 61, 62]. However, neither TLR4-signaling [33] nor the carboxy-terminal tail of the protein [62] is needed for the internalization. After internalization, the TLR4/MD2/CD14 complex can subsequently be detected in the early endosomal compartment defined by the EEA1 and Rab5 markers [25, 41]. The Rab7b [63] and Rab11a proteins [64] regulate the further cytosolic sorting of TLR4/CD14.

In analogy with these previous findings we show herein that the focal S100A9-binding detected both on the plasma membrane and in cytosolic vesicles of TLR4-KO cells, co-localized with CD14 expression. Further, we show that the S100A9 co-localized with Rab5 in such vesicles and there was no detectable internalization of S100A9 into CD14-KO cells. Thus, similarly to LPS, the internalization of S100A9 is CD14-dependent. The TLR4/MD2/CD14 complex is known to recycle from the plasma membrane to Golgi apparatus [35, 65]. While this mechanism is not essential for TLR4 signaling, LPS was shown to follow that route of recirculation upon stimulation of TLR4 [35, 66]. In our experiments we could also detect gold-labeled huS100A9 in association with the Golgi apparatus and also with rough endoplasmic reticulum in the cytosol of THP-1 cells (**S2D Fig**). The finding of S100A9 association with the Golgi apparatus suggests that also in this respect S100A9-mediated TLR4-stimulation may follow the same general pathway as stimulation by LPS.

Most importantly, we could show that the co-localization of CD14 and S100A9 proteins is functionally relevant. Thus, the S100A9-induced cytokine response was clearly CD14-dependent as it was eliminated in CD14-KO BM-DCs. That finding was further supported by the experiments showing that CD14 antibodies blocking LPS-induced TLR4 stimulation, could also block S100A9-induced TLR4-stimulation in human THP-1 cells. These data provided functional data strongly supporting the hypothesis that CD14 also acts as a co-receptor in the S100A9-induced response. Further, we could detect specific binding of S100A9 to CD14 in SPR analyses, but failed to detect specific binding of huS100A8 to CD14. Our previous paper showed that huS100A8 binds less well than huS100A9 to TLR4 [24]. Taken together, these results indicate that CD14 is a co-receptor for the S100A9-induced stimulation of TLR4.

Unexpectedly, we could detect binding of S100A9 to the surface of CD14-KO BM-DCs, indicating that these cells express other S100A9-binding receptors as well. The BM-DCs were generated by culturing BM cells in the presence of GM-CSF. BM-DCs most probably originate from monocytes and these cells express lower level of cell surface CD14 than macrophages [33, 67]. The integrin CD11b, also known as complement receptor 3 (CR3), has been shown to facilitate the uptake of LPS in BM-DCs and myeloid DCs [67]. Further, several other membrane proteins CD85j [68], CD147 [69] and CD33 [70] have been shown to be receptors for S100A9. At present we do not know the nature of the S100A9-binding detected on the CD14-KO BM-DCs, but these previously described receptors are potential candidates since they are all expressed in monocytoid cells. However, deletion of either TLR4 or CD14 was sufficient to completely inhibit the S100A9-induced cytokine response, defining these as essential receptors of that response.

While several previous studies [33, 61, 62] have shown that the CD14 protein is essential for LPS-induced internalization of TLR4, a recent study provided evidence indicating that both an agonistic TLR4/MD2 specific antibody and a small synthetic TLR4 ligand could induce CD14-independent internalization and endosomal TLR4 signaling [71]. Thus, at least some ligands can cause internalization of TLR4/MD2 through a CD14-independent pathway. Upon ligand-binding MD2 was shown to promote the dimerization and internalization TLR4/MD2 [62, 72]. Both the TLR4/MD2-specific antibody and the synthetic TLR4-ligand used by Rajaiah et al [71] could potentially cause dimerization of TLR4/MD2 and thereby induce the CD14-independent internalization. As shown here, however, the stimulation of BM-DCs with S100A9 is both TLR4- and CD14-dependent. In addition, we did not detect internalization of the S100A9 protein in CD14-KO cells, suggesting that S100A9, similarly to LPS, may induce CD14-dependent internalization of TLR4/MD2.

We used recombinant huS100A9 produced in E. coli bacteria in our experiments and it was important to take precautions to avoid the involvement of LPS [73] in the cytokine response induced upon stimulating monocytes with the protein. Thus, LPS contaminants were removed by affinity chromatography during S100A9 protein preparation and addition of polymyxin B to stimulation cultures could confirm that induced cytokine responses were largely insensitive to this compound. Additionally, the CD14 blockade with specific antibodies strongly reduced the S100A9-induced cytokine response, while it did not affect the response induced by the TLR2-agonist Pam_3_Cys, indicating that bacterial TLR2 stimulators do not contaminate the protein either.

Taken together, we show herein that CD14 is an essential co-receptor of the S100A9-induced cytokine response in monocytoid cells. Our findings further indicate that while CD14 can bind S100A9 and may be essential for the endocytosis of the S100A9 protein, there are other putative S100A9 receptors present on the surface of such cells. The identity of these putative receptors is currently unknown.

## Supporting Information

S1 Fig (A) S100A9 co-localizes with TLR4 in cytosolic vesicles. THP-1 cells were prepared as in Fig 2B. (B) Vesicular co-localization of S100A9 and cav-1 in TLR4-KO BM-DCs. Same specimen as in Fig 4B.
(TIF)

S2 Fig (A) BM-DCs from TLR4-KO mice were incubated with gold-labeled S100A9 (10nm grains) as in Fig 2. The specimen was thereafter immuno-stained with rat anti-mouse CD14 antibody, followed by gold-labeled (25nm grains) secondary antibody. The image shows representative sites of surface and vesicular binding of the S100A9-protein and CD14-expression. Bar: 500nm. (B) Parallel cultures stimulated as those in Fig 5C were exposed to polymyxin B and the TNFα-response analyzed. (C) BM-DCs from TLR4-KO mice pre-incubated with anti-CD14 antibodies as in Fig 5B and subsequently incubated with gold-labeled S100A9 (10nm grains) as in Fig 2. The image shows representative sites of surface binding of the S100A9-protein. Bar: 500nm. (D) Co-localization of S100A9 with Golgi apparatus (lower right quadrant) and rough ER (upper right quadrant) in THP-1 cells. Specimen was prepared as in Fig 2A. Bar: 500nm
(TIF)
