# Supplementary figures and images for: CD14 Is a Co-Receptor for TLR4 in the S100A9-Induced Pro-Inflammatory Response in Monocytes
Source: PLoS One. 2016 May 26;11(5):e0156377. doi: 10.1371/journal.pone.0156377 (PMC4881898; doi:10.1371/journal.pone.0156377)

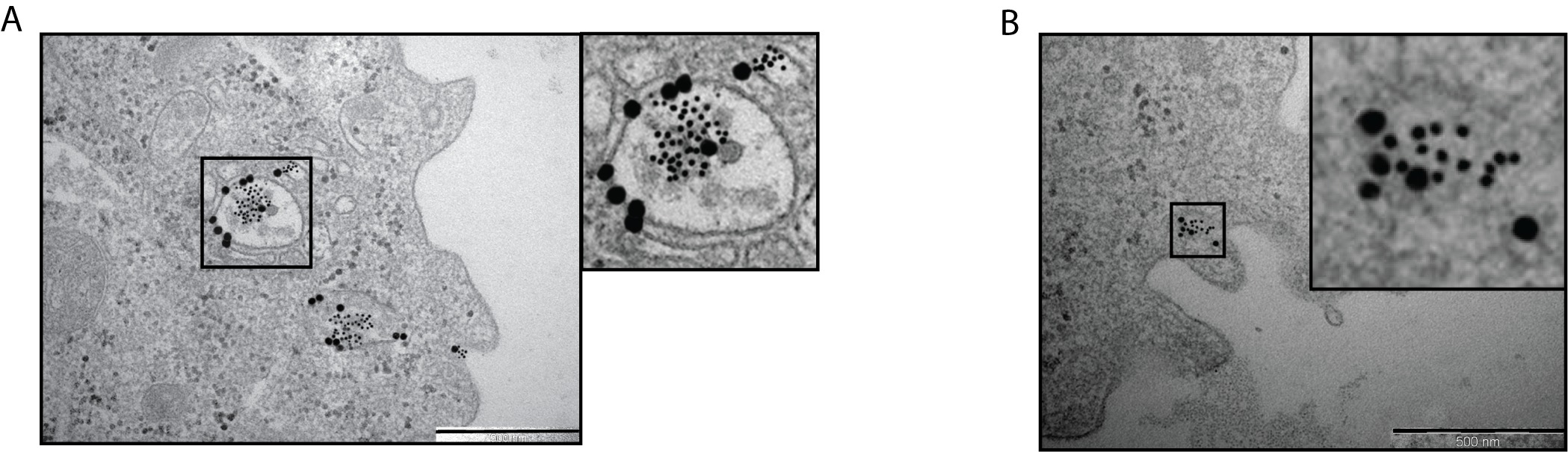

Supplement: S1 Fig — (A) S100A9 co-localizes with TLR4 in cytosolic vesicles. THP-1 cells were prepared as in Fig 2B. (B) Vesicular co-localization of S100A9 and cav-1 in TLR4-KO BM-DCs. Same specimen as in Fig 4B. (TIF) [file pone.0156377.s001.tif]

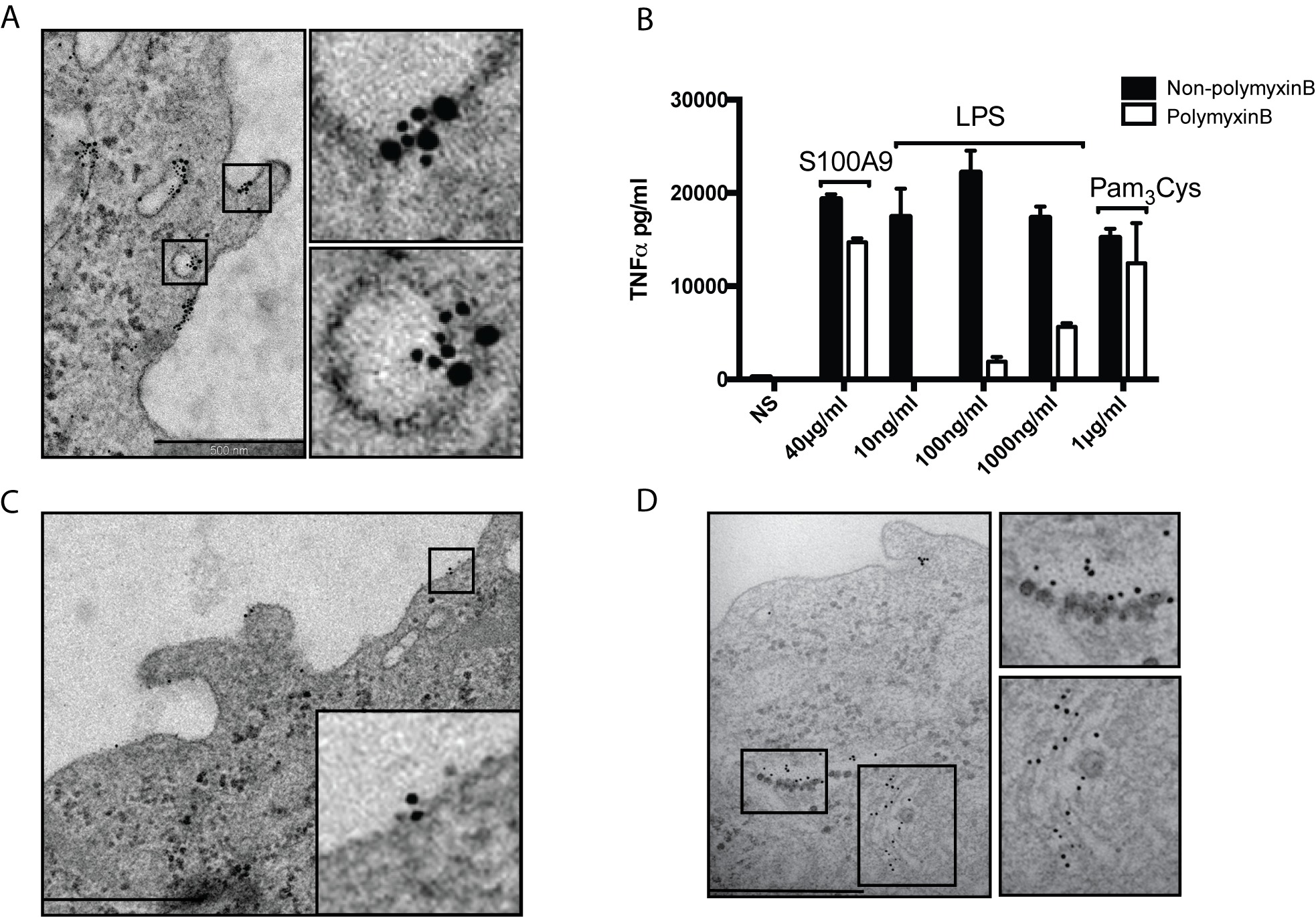

Supplement: S2 Fig — (A) BM-DCs from TLR4-KO mice were incubated with gold-labeled S100A9 (10nm grains) as in Fig 2. The specimen was thereafter immuno-stained with rat anti-mouse CD14 antibody, followed by gold-labeled (25nm grains) secondary antibody. The image shows representative sites of surface and vesicular binding of the S100A9-protein and CD14-expression. Bar: 500nm. (B) Parallel cultures stimulated as those in Fig 5C were exposed to polymyxin B and the TNFα-response analyzed. (C) BM-DCs from TLR4-KO mice pre-incubated with anti-CD14 antibodies as in Fig 5B and subsequently incubated with gold-labeled S100A9 (10nm grains) as in Fig 2. The image shows representative sites of surface binding of the S100A9-protein. Bar: 500nm. (D) Co-localization of S100A9 with Golgi apparatus (lower right quadrant) and rough ER (upper right quadrant) in THP-1 cells. Specimen was prepared as in Fig 2A. Bar: 500nm (TIF) [file pone.0156377.s002.tif]
